# Ecrg4 peptide is the ligand of multiple scavenger receptors

**DOI:** 10.1038/s41598-018-22440-4

**Published:** 2018-03-06

**Authors:** Tetsuo Moriguchi, Shuji Takeda, Shinzo Iwashita, Kei Enomoto, Tatsuya Sawamura, Uichi Koshimizu, Toru Kondo

**Affiliations:** 1 Division of Stem Cell Biology, Institute for Genetic Medicine, Hokkaido University, Sapporo, Hokkaido, 060-0815 Japan; 2 ASUBIO Pharma Co., Ltd., Kobe, Hyogo, 650-0047 Japan; 3 Department of Physiology, Shinshu University School of Medicine, 3-1-1 Asahi, Matsumoto, 390-8621 Japan

## Abstract

Esophageal cancer-related gene 4 (*Ecrg4*) encodes a hormone-like peptide that is believed to be involved in a variety of physiological phenomena, including tumour suppression. Recent progress in the study of Ecrg4 has shown that Ecrg4 is a proinflammatory factor and induces the expression of several cytokines and chemokines in macrophages/microglia. However, the detailed molecular mechanisms of Ecrg4 signalling, especially the Ecrg4 receptors, remain poorly understood. Here, using retrovirus-mediated expression cloning, we identified lectin-like oxidised low-density lipoprotein receptor-1 (LOX-1) as a membrane protein that binds amino acid residues 71–132 of Ecrg4 (Ecrg4(71–132)). Moreover, in addition to LOX-1, several scavenger receptors, such as Scarf1, Cd36 and Stabilin-1, facilitated the efficient internalisation of Ecrg4(71–132) into cells. A broad competitive inhibitor of scavenger receptors, polyinosinic acid, reduced both the binding of Ecrg4(71–132) and the activation of NF-κB in microglia. This activation was dependent on MyD88, an adaptor protein that recruits signalling proteins to Toll-like receptors (TLRs), with the consequent induction of various immune responses. These data suggest that multiple scavenger receptors recognise Ecrg4(71–132) and transduce its signals, together with TLRs, in microglia.

## Introduction

Peptide hormones regulate many biological processes, and in many cases, their biological information is transduced into the target cells through specific receptors. The esophageal cancer-related gene 4 (Ecrg4) product, also known as augurin, is a recently identified neuronal peptide hormone that was originally reported as a candidate tumour suppressor^1,2^. Since then, ECRG4 has been shown to participate in many physiological phenomena, including tumour suppression, cell senescence, central nervous system homeostasis, Alzheimer’s disease, and immune responses^3–12^.

*Ecrg4* is conserved from fish to humans, and mammalian *Ecrg4* encodes a precursor protein of 148 amino acids. Several post-translational modifications of Ecrg4 have been reported, and the most important is its proteolytic processing^2,13,14^. The first 30 amino acids of Ecrg4 function as a signal peptide and are cleaved off in the secretion pathway^14^. A conserved consensus sequence for furin, a member of the prohormone convertases, is located in the middle region of Ecrg4, and a thrombin cleavage site occurs near the C-terminus^2,13^. Thus, like other neuronal peptide hormones, the Ecrg4 precursor protein produces several different peptides. Although several reports have shown that Ecrg4 inhibits cell proliferation, accumulating evidence including ours indicates proinflammatory functions for the Ecrg4 peptides: cerebral ventricular injection of amino acids 71–148 of Ecrg4 increased plasma adrenocorticotropic hormone and corticosterone in rats^15^. The C-terminal Ecrg4 peptide containing amino acids 133–148 (Ecrg4(133–148)) activated the NF-κB pathway in macrophages and interacted with the innate immunity receptor complex (Toll-like receptor 4 (TLR4)/CD14/MD2 complex)^16,17^. Ecrg4(71–132) and Ecrg4(133–148) activated macrophages/microglia and induced the expression of several cytokines and chemokines, which contribute, at least in part, to the antitumour function of Ecrg4^10,11^. Together, these findings suggest that Ecrg4 acts as an inflammatory cytokine that regulates many physiological processes, rather than a traditional tumour suppressor. To unravel the molecular mechanisms of the Ecrg4 signalling pathways, we searched for Ecrg4 receptors in this study.

## Results

### Identification of LOX-1 as an Ecrg4-binding membrane protein

To identify the Ecrg4 receptor, we generated a mouse Ecrg4–human Fc fusion protein (Ecrg4–Fc) in HEK293 cells. We found that the conditioned medium contained several bands that reacted with anti-Ecrg4 and anti-human Fc antibodies (Supplementary Fig. S1), although Ecrg4 was shown to be translocated to the cell surface and not released into the culture medium in HEK293 cells^14^. These mixed Ecrg4–Fc proteins were used for subsequent receptor screening.

**Figure 1:**
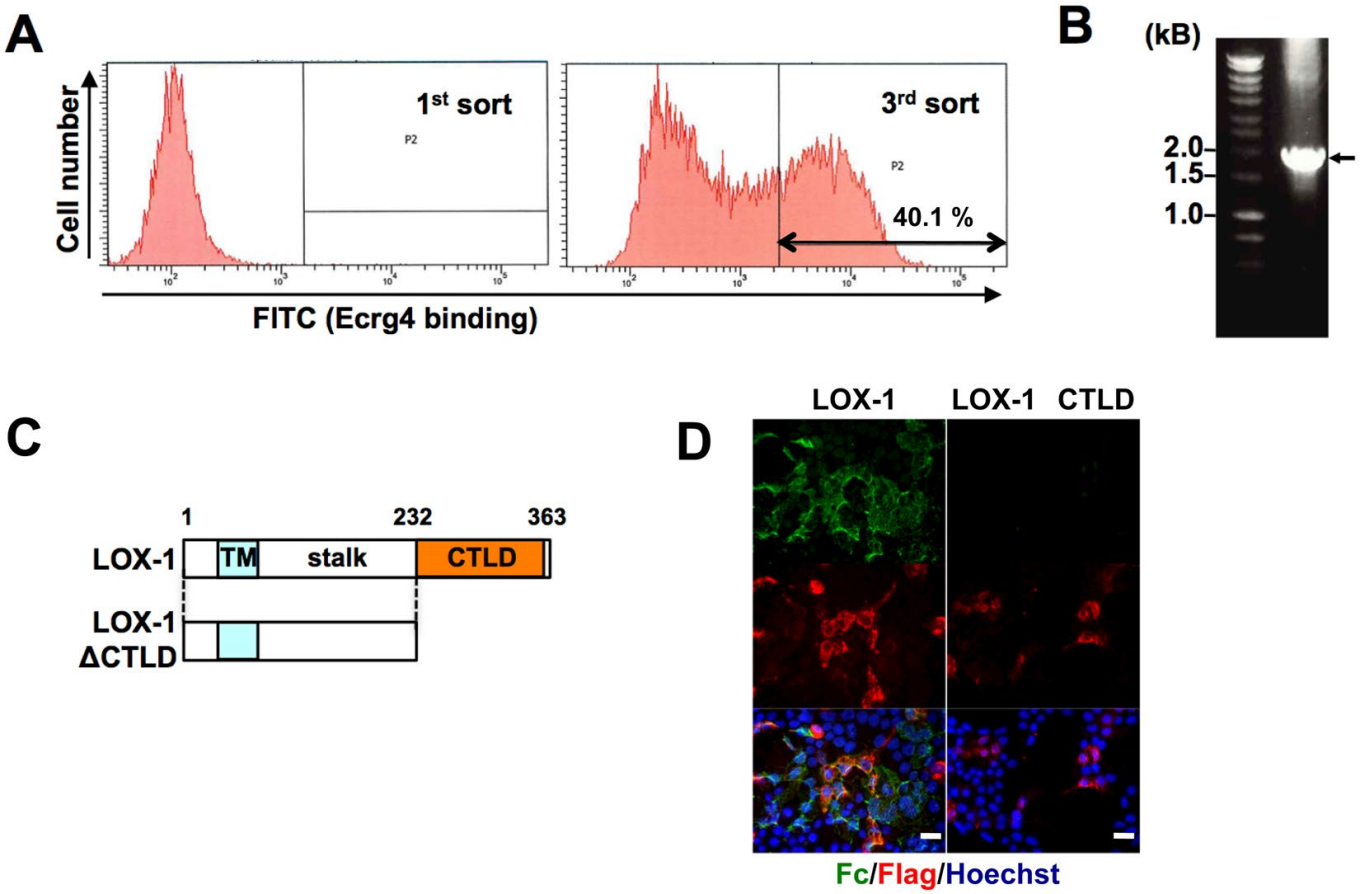
Molecular cloning of LOX-1. (**A**) Ba/F3 cells were retrovirally transfected with a cDNA library from the variant Ba/F3 (#BEB1) cell line, immunologically stained for Ecrg4–Fc, sorted with a fluorescence-activated cell sorter, expanded in culture, and sorted again. The Ecrg4–Fc staining profiles of the Ba/F3 cells (1^st^ sort) and the cells after two cycles of sorting (3^rd^ sort) are shown. (**B**) The genes derived from the cDNA library were amplified with PCR using single-cell clones stained for Ecrg4–Fc from the P2 area of the 3^rd^ sorted cells. The amplified fragment (indicated by an arrow) was sequenced. LOX-1 cDNA, including the full-length coding region, was isolated from all three single clones. (**C**) Schematic representation of mouse LOX-1 and its truncated mutant. TM, transmembrane domain; CTLD, C-type lectin-like domain. (**D**) HEK293 T cells transfected with Flag-tagged wild-type mouse *LOX-1* (left) or a CTLD-deletion mutant of *LOX-1* (right) were incubated with Ecrg4–Fc before cell fixation. The binding of Ecrg4–Fc was detected with a FITC-labelled anti-human IgG Fc antibody (green; Fc), and the Flag-tagged LOX-1 expression was detected with an anti-Flag antibody (red; Flag). Nuclei were stained with Hoechst (blue). Scale bar 20 μm.

We constructed a retrovirus-based expression library^18^ prepared from the cDNA of CG4 cells and infected Ba/F3 cells with the virus. Using the Ecrg4–Fc protein, we sorted Ecrg4–Fc-binding cells by flow cytometry and expanded them. After 5 rounds of this sorting and expansion cycle, we obtained a single clone (designated BaF/3 (#BEB1) thereafter), which acquires a high binding capacity for Ecrg4 (Supplementary Fig. S2). We further prepared an expression library from BaF/3 (#BEB1) mRNA and introduced the library into the parental Ba/F3 cells. After three cycles of sorting and expansion, we observed that over 40% of the cells were strongly bound to Ecrg4 (Fig. 1A). After we obtained Ecrg4–Fc-binding single-cell clones, we identified LOX-1, which was commonly expressed in most of the clones, using retrovirus-specific primers (Fig. 1B). LOX-1 is a type II transmembrane protein with a C-type lectin-like domain (CTLD) in the extracellular region, which is required for the recognition of its ligands, such as ox-LDL^19,20^ and see (Fig. 1C). The results of a microarray indicated that the *LOX-1* mRNA levels were much higher in the BaF/3 (#BEB1) cells than the parental Ba/F3 cells (Supplementary Fig. S2).

To confirm that Ecrg4 binds LOX-1, we incubated LOX-1-overexpressing HEK293 T cells with Ecrg4–Fc to demonstrate the interaction between LOX-1 and ox-LDL^21^. We verified that Ecrg4–Fc clearly bound to the LOX-1-expressing cells, whereas it did not bind to the cells expressing CTLD-deleted LOX-1, revealing that CTLD is the Ecrg4-binding domain.

### LOX-1 recognises residues 71–132 of Ecrg4

**Figure 2:**
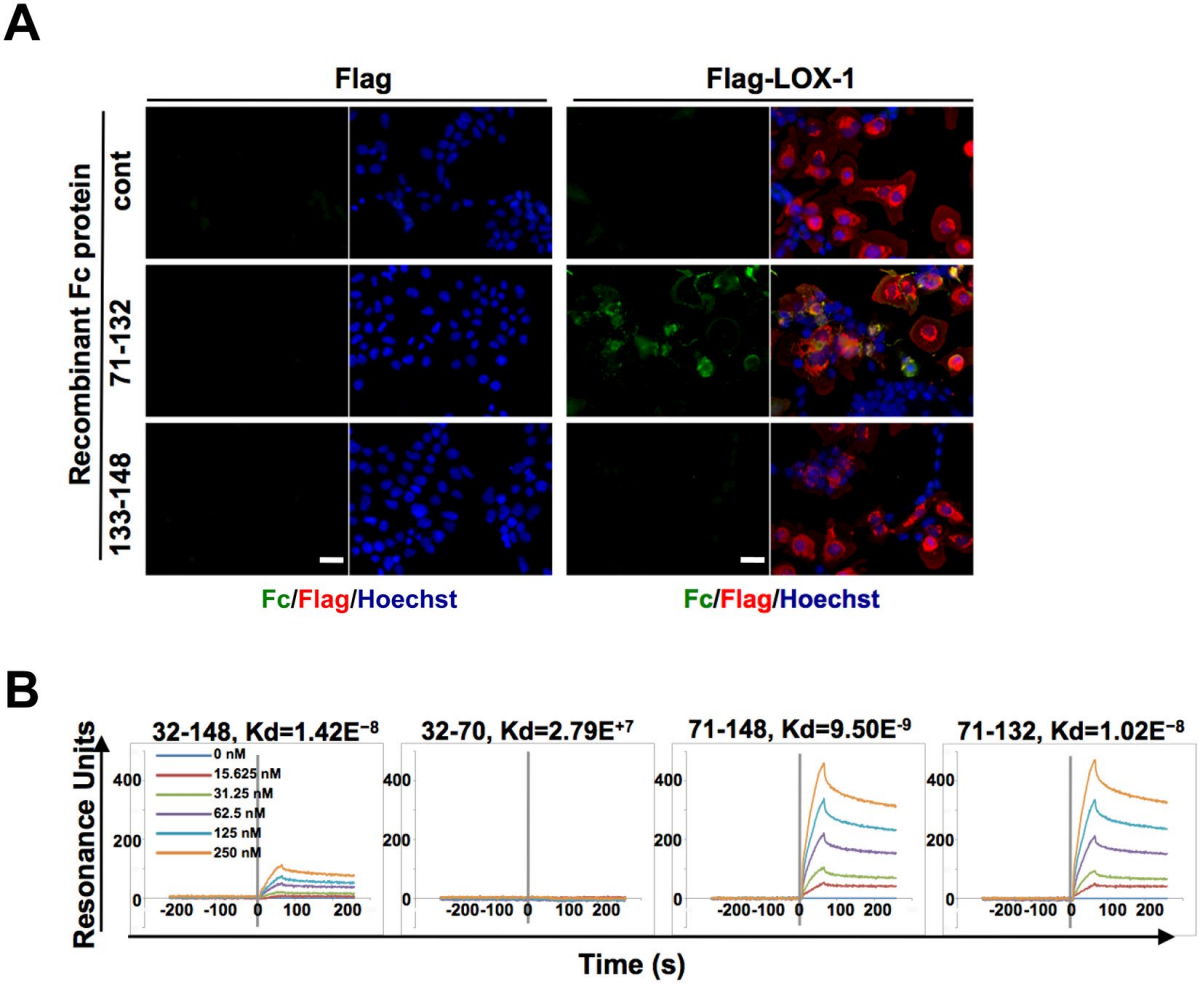
LOX-1 is a binding partner of Ecrg4(71–132). (**A**) HEK293 T cells were transfected with Flag-tagged *LOX-1* and incubated with Ecrg4–Fc deletion mutants (Ecrg4(71–132) and Ecrg4(133–148)) before cell fixation. The binding of the Ecrg4–Fc deletion mutants was detected with a FITC-labelled anti-human IgG Fc antibody (green), and the Flag-tagged LOX-1 expression was detected with an anti-Flag antibody (red). Nuclei were stained with Hoechst (blue). (**B**) SPR analysis of the interaction between LOX-1 CTLD and ECRG4 or its deletion mutants. C-terminally His-tagged ECRG4(32–148), ECRG4(32–70), ECRG4(71–148), and ECRG4(71–132) were immobilised on a sensor chip with an anti-His-tag antibody. The sensorgrams were measured after the injection of the Fc-fused 2 × CTLD of LOX-1 at concentrations of 15.625, 31.25, 62.5, 125, and 250 nM. The calculated Kd (dissociation constant, in M) for each pair is also shown.

The Ecrg4 protein is cleaved by peptidases, such as furin and thrombin, and processed into smaller peptide fragments. Among them, at least two fragments, amino acids 71–132 and 133–148, have been shown to induce the expression of cytokines and chemokines^10,11^. To determine which Ecrg4 peptide binds to LOX-1, we incubated LOX-1-expressing cells with the recombinant Ecrg4(71–132)–Fc and Ecrg4(133–148)–Fc proteins. As shown in Fig. 2A, Ecrg4(71–132) attached to the LOX-1-expressing cells, whereas Ecrg4(133–148) did not.

To measure the avidity of Ecrg4 peptides to LOX-1, we performed a surface plasmon resonance (SPR) analysis. We prepared four human ECRG4 peptides (ECRG4(32–148), ECRG4(32–70), ECRG4(71–148) and ECRG4(71–132)) with a 6 × His-tag. Because the oligomerisation of LOX-1 is required for binding with its ligands, we prepared 2 × CTLD–Fc protein as reported by Cao *et al*.^22^. As shown in Fig. 2B, ECRG4(32–148), ECRG4(71–148) and ECRG4(71–132) produced significant increases in the resonance units (RU), whereas ECRG4(32–70) did not. The dissociation constants (Kd) for ECRG4(32–148), ECRG4(71–148) and ECRG4(71–132) with 2 × CTDL were almost the same, approximately 1 × 10^−8^ M. These results indicated that ECRG4(71–132) directly and strongly binds to the CTLD of LOX-1.

### Multiple scavenger receptors act as receptors for Ecrg4(71–132)

In our previous work, we identified Ecrg4(71–132) as an inducer of several inflammatory cytokines in primary microglia prepared from mouse pups^11^. To examine whether the Ecrg4(71–132)-induced cytokine production in microglia is dependent on LOX-1, we prepared microglia from *LOX-1* knockout (*LOX-1*^−/−^) mice and investigated their reactivity against the Ecrg4 peptide. Unfortunately, Ecrg4(71–132) bound *LOX-1*^−/−^ microglia and induced the expression of inflammatory cytokines (Supplementary Fig. S3 and data not shown), suggesting that other unknown receptors for Ecrg4(71–132) exist on microglia.

**Figure 3:**
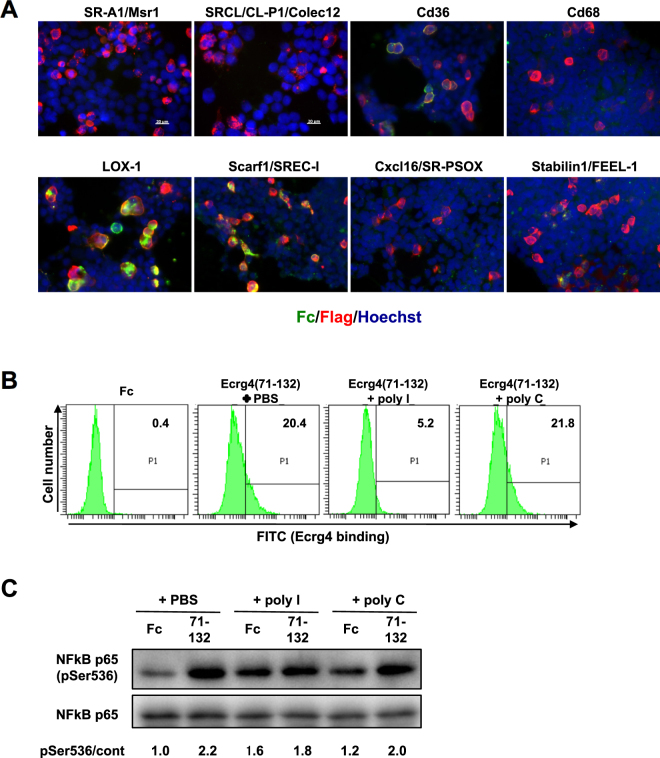
Internalisation of Ecrg4(71–132) and activation of NF-κB by Ecrg4(71–132) via several scavenger receptors. (**A**) HEK293 T cells were transfected with several Flag-tagged scavenger receptors. Two days after transfection, the cells were treated with Ecrg4(71–132) for 2 h. Ecrg4(71–132)–Fc was detected with a FITC-labelled anti-human IgG Fc antibody (green) and the transiently expressed Flag-tagged receptors were detected with an anti-Flag antibody (red). Nuclei were stained with Hoechst (blue). (**B**) Fluorescence intensities of Ecrg4(71–132) bound to primary microglia. Primary microglia were incubated with Fc(N293A)-fused Ecrg4(71–132) for 1 h in the presence of PBS, polyI, or polyC. (**C**) Immunoblotting for p65 in microglial cells treated with Ecrg4(71–132) in the presence of PBS, polyI, or polyC. The band intensity was quantified by a ChemiDoc™ MP Imaging system (Bio-Rad), and the relative band intensity ratio NF-κB p65(pSer536)/NF-κB p65 is shown (pSer536/cont). Representative data from three independent experiments are indicated.

Since the CTLD in LOX-1 is essential for Ecrg4(71–132)-binding (Figs 1D and 2B), we examined whether Ecrg4(71–132) binds to other CTLD-containing receptors (CLRs). None of the CLRs tested bound Ecrg4(71–132) in the cultured-cell-based binding assay (Supplementary Fig. S4). We then addressed whether Ecrg4(71–132) bound to the scavenger receptors consisting of 7 classes (A, B, D, E, F, G and H), because LOX-1 belongs to a receptor family that is defined by the binding capability of modified LDLs and their internalisation^23,24^. As shown in Fig. 3A and Supplementary Fig. S5, Scarf1/endothelial cell-I (SREC-I), Cd36 and Stabilin-1/FEEL-1 (fasciclin, epidermal growth factor (EGF)-like, laminin-type EGF-like, and link domain-containing scavenger receptor-1), members of the F, B and H classes respectively, efficiently internalised Ecrg4(71–132). Indeed, several scavenger receptors including LOX-1, Cd36, and Stabilin-1/FEEL-1 are highly expressed in primary microglia. Because many classes of scavenger receptors recognised Ecrg4(71–132), we examined the effect of polyinosinic acid (polyI), a broad competitive inhibitor of scavenger receptors, which has been shown to inhibit the functions of LOX-1, Scarf/SREC-I, and Stabilin1/FEEL-1^25–29^. PolyI, but not polycytidylic acid (polyC), inhibited the binding of Ecrg4(71–132) and the phosphorylation of p65 in microglial cells, although treatment of polyI itself slightly increased the p65 phosphorylation (Fig. 3B,C). These data suggest that Ecrg4(71–132) binds to several scavenger receptors and activates NF-κB signalling.

### Activation of NF-κB by Ecrg4(71–132) is dependent on the MyD88 adaptor protein

**Figure 4:**
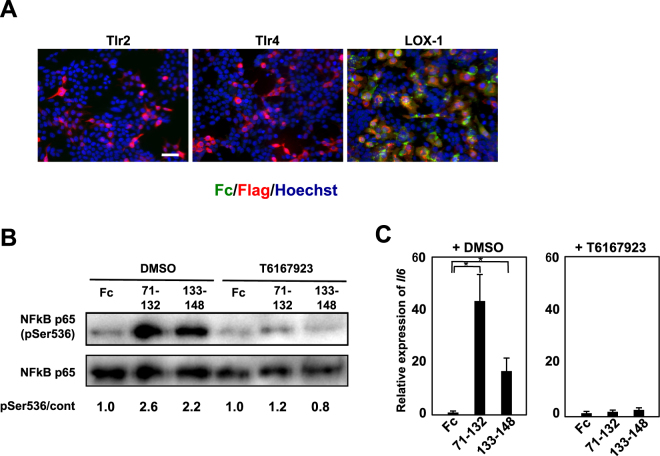
Ecrg4 activates NF-κB signalling via MyD88. (**A**) HEK293 T cells expressing Tlr2, Tlr4, or LOX-1 were incubated with Ecrg4(71–132)–Fc for 2 h and immunostained as in Fig. 3A. Scale bar: 50 μm. (**B**) MyD88 inhibitor T6167923 was added to the culture medium 1 h before stimulation with Ecrg4. After stimulation for 30 min with 20 µg/mL of Fc, Ecrg4(71–132)–Fc, or Ecrg4(133–148)–Fc, the extracts of microglial cells were immunoblotted for p65. The relative band intensity ratio NF-κB p65(pSer536)/NF-κB p65 is shown (pSer536/cont) as in Fig. 3C. Representative data from three independent experiments are indicated. (**C**) Primary microglial cells were stimulated with 20 µg/mL of Fc, Ecrg4(71–132)–Fc, or Ecrg4(133–148)–Fc for 3 h. The mRNA levels of *Il6* were evaluated with quantitative RT–PCR and are shown as relative expression. The means and SDs of the data from three independent experiments are shown. *p < 0.05, compared with Fc, two-sided Student’s t test.

Previous studies have reported that several scavenger receptors recognise danger-associated molecular patterns (DAMPs) and pathogens^23,24^ and trigger a proinflammatory response in combination with members of the TLR family. Since Ecrg4(133–148) has been shown to interact with TLR4, a member of the TLR family^17^, we investigated the involvement of TLRs in the function of Ecrg4(71–132). We overexpressed either TLR4 or TLR2 in HEK293 T cells, incubated with Ecrg4(71–132), and tested its internalisation into the receptor-expressing cells. As shown in Fig. 4A, we found that neither TLR4 nor TLR2 internalised Ecrg4(71–132) as effectively as LOX-1 did. To clarify the role of TLRs in Ecrg4 signalling, we used T6167923, a chemical inhibitor for MyD88 that is essential for the TLR signalling pathway^30^. Microglia were preincubated with T6167923, stimulated with Ecrg4(71–132), and examined for p65 phosphorylation and *Il6* expression. The blockade of MyD88 abolished both the phosphorylation of p65 and the increased expression of *Il6* (Fig. 4B and C). These results suggested that TLRs have important roles in Ecrg4(71–132) signalling, although they did not associate with Ecrg4(71–132).

## Discussion

In this study, we demonstrated for the first time that Ecrg4(71–132) binds to multiple scavenger receptors that activate the innate immune responses^24^ and maintain homeostasis by clearing modified lipoproteins, pathogens and apoptotic cells^23^. These results are consistent with our previous finding that Ecrg4 peptides induce the expression of proinflammatory factors and contribute to anti-glioma immunosurveillance^11^. The diverse combination of scavenger receptors, their ligands and co-factors generates the functional versatility via a variety of intracellular signalling pathways. For example, LOX-1, which binds with ox-LDL, rapidly increased reactive oxygen species levels and activated the NF-κB signalling pathway^31,32^, while with the outer membrane protein A (OmpA), LOX-1 triggered the innate immune response via a TLR2-dependent pathway^33^. Our findings suggest that scavenger receptors, in cooperation with MyD88 dependent molecules, such as TLR family receptors, activate the Ecrg4(71–132) signal pathway, similar to the activation of pathogen-triggered scavenger receptors^33–37^. We observed that Ecrg4-Fc did not activate NF-κB signaling in BaF/3 #BEB1 and 293 T cells that overexpressed LOX-1 alone, LOX1 and Tlr2, or LOX-1 and Tlr4, although it was internalised into the cells (data not shown). These results indicated that the scavenger receptors are essential for internalising the peptide but not for initiating signal transduction, and that LOX-1 and unknown factor are needed for Ecrg4-dependent activation of NF-κB signaling. Interestingly, we found that Ecrg4(133–148), which differs from Ecrg4(71–132) in its recognition by scavenger receptors examined in this manuscript, also induced the expression of cytokines via the MyD88-NF-κB signalling axis (Fig. 4B,C), suggesting that it transmits inflammatory signals through an unidentified component/receptor, although Ecrg4(133–148) was shown to interact with the TLR4/CD14/MD2 complex^17^. Thus, the detailed molecular mechanisms of Ecrg4 peptides should be determined in future studies.

The scavenger receptors recognise a wide range of ligands. For example, modified lipoproteins, polyanions (polyI and others), C-reactive protein (CRP), heat shock protein, apoptotic cells and bacteria bind to LOX-1 through the CTLD^20^. In this study, we showed that Ecrg4(71–132) bound to the CTLD of LOX-1. The calculated Kd value for the binding of ECRG4 and LOX-1 is similar to those for ox-LDL and CRP. The Kd for ox-LDL and LOX-1 was 1.7 × 10^−8^ M when measured in living cells^38^ and 1.8 × 10^−10^ M when measured from the clustered CTLDs on a sensor chip^39^. The Kd for CRP and LOX-1 is reportedly 1.6 × 10^−7^ M^40^. The crystal structure of the CTLD of LOX-1 has already been investigated^41^, and thus, the *in silico* characterisation of the binding mode or a structural analysis of the CTLD when complexed with short peptides may clarify the features of this ligand binding reaction. Scavenger receptors are also considered a subclass of the pattern recognition receptors because they recognise not only pathogen-associated molecular patterns (PAMPs) but also modified lipids and proteins that are considered DAMPs^23,24^. Ecrg4 peptides are endogenous proteins, and therefore, they should be classified as DAMPs. Investigation of the regulatory mechanisms of *Ecrg4* gene expression may provide insight into Ecrg4 functions and cellular danger signals.

In conclusion, we demonstrated that Ecrg4 interacts directly with LOX-1 and is internalised via several scavenger receptors. Since combination and mechanisms of Ecrg4/receptors may be different in each physiological phenomena and disease, therefore, the detailed mechanisms should be solved in future studies. Our findings not only support the roles of Ecrg4 in tissue homeostasis and the inflammatory response but should promote further analyses of the physiological functions of Ecrg4 and the pathogenesis of multiple diseases.

## Methods

### Cells and animals

HEK293 T cells and Plat-E cells were cultured in Dulbecco’s modified Eagle’s medium (DMEM) containing 10% foetal calf serum (FCS). The rat oligodendrocyte precursor cell line CG4 was grown as described previously^5^. The Ba/F3 cells and a variant strain of BaF/3, BaF/3 (#BEB1) cells, which had an increased capacity to bind Ecrg4, were maintained in RPMI medium containing 10% FCS and 10 ng/mL recombinant mouse IL-3 (PeproTech). C57BL/6 mice were purchased from CLEA Japan. All mouse experiments were performed following the protocols approved by the Animal Care and Use Committee of Ehime University and Hokkaido University. Primary microglial cells were isolated from mouse pups as previously described^42^.

### Plasmids and other reagents

The DNA fragment encoding full-length mouse Ecrg4 was introduced into the pEF-Fc plasmid kindly provided by Dr. Nagata and used to obtain a mixture of the processed forms of the Ecrg4–Fc proteins. The expression vectors for Fc-fused Ecrg4(71–132) and Ecrg4(133–148) have been described previously^11^. For production of the C-terminal 6 × His-tagged proteins for SPR analysis, fragments of human *ECRG4* of varying lengths (encoding residues 32–148, 32–70, 71–148, or 71–132) were generated with PCR and cloned into the *Nde*I and *Xho*I sites of pET30a (Novagen). Mouse LOX-1/Olr-1 (NM_138648), Msr1/SR-A (NM_031195), Cd36 (NM_007643), Colec12/CL-P1 (NM_130449), Scarf1/SREC-I (NM_001004157), Cd68 (NM_001291058), Stabilin1/FEEL1 (NM_138672), Tlr2 (NM_011905), and Tlr4 (NM_021297) cDNAs were isolated from the variant Ba/F3 (#BEB1) cells or the spleens of C57BL/6 mice. A truncated mouse LOX-1 mutant (ΔCTLD); 2 × CTLD–Fc, which included two human LOX-1 CTLDs in tandem; and mutants of the Fc tag, Fc(N297A), containing an asparagine (N) to alanine (A) substitution at original position 297, were constructed with PCR-based techniques. T6167923^29^ was purchased from Aobious Inc. PolyI and polyC were purchased from Santa Cruz Biotechnology.

### Recombinant proteins

For construction of the Fc fusion proteins, HEK293 T cells were transfected with the appropriate expression constructs using polyethylenimine (PEI). Two days after transfection, the conditioned medium was collected, centrifuged, and passed through a 0.45 µm filter membrane. The Fc fusion proteins were purified with Protein A–Sepharose 4 Fast Flow (GE Healthcare) and assessed with sodium dodecyl sulphate-polyacrylamide gel electrophoresis (SDS-PAGE), Coomassie Brilliant Blue (CBB) staining, and immunoblotting. For the microglial experiments, Fc (N297A) fusion proteins were produced to minimise the effect of the endogenous Fc receptors^43^. His-tagged proteins were produced as inclusion bodies in *Escherichia coli* strain BL21(DE3) and purified under denaturing conditions with cOmplete™ His-Tag Purification Resin (Roche). After dialysis against phosphate-buffered saline (PBS), the supernatants were used for subsequent experiments.

### Retrovirus-based cDNA expression library

Poly(A)^+^ mRNA was purified with a mRNA purification Kit (QuickPrep Micro mRNA Purification Kit; GE Healthcare). Double-stranded cDNA was synthesised with a cDNA synthesis kit (SuperScript Choice System for cDNA Synthesis; Thermo Fisher Scientific), and a *Bst*XI adaptor was attached. The fragments were purified, size-fractionated with spin columns (Chroma Spin™−400; Clontech), and ligated into the *Bst*XI-digested pMYs vector. Plat-E cells were transfected with the plasmid DNA from the cDNA library using PEI. Two days after transfection, the culture supernatants were collected and used for viral infection. The infected Ba/F3 cells were incubated with Ecrg4–Fc on ice for 20 min and then with fluorescein isothiocyanate (FITC)-conjugated F(ab’)2 donkey anti-human IgG Fc (Jackson ImmunoResearch) and were then sorted on a FACSAria™ II flow cytometer (BD Biosciences). For isolation of the cDNA integrated into the retroviral vector, the genomic DNA was extracted from transformed Ba/F3 cells with standard methods and subjected to PCR with Ex Taq DNA Polymerase (TaKaRa) and PCR primers (5′-CTCTAGACTGCCGGATCTAGCTAGT-3′ and 5′-CTATGGCTCGTACTCTATAGGCTTC-3′) carrying sequences from the pMYs vector.

### Immunoblotting analysis

Cells were stimulated with Ecrg4(71–132)–Fc or Ecrg4(133–148)–Fc in the presence of the indicated chemical reagents for 30 min and washed twice with ice-cold PBS. Whole-cell extracts were obtained by sonicating the cells in 1 × SDS sample buffer. SDS-PAGE and electrotransfer were performed with standard methods. The membranes were probed with antibodies against phospho-p65 (pSer536) and p65 (Cell Signaling Technology) overnight at 4 °C and then with horseradish-peroxidase-conjugated anti-rabbit IgG (GE Healthcare). Clarity Western ECL Substrate (Bio-Rad) was used for detection. In some case, the membranes were treated with stripping buffer (2% SDS, 100 mM 2-mercaptoethanol, 62.5 mM Tris-HCl, (pH 6.8)) to investigate phospho-p65 (pSer536) and p65 on the same blot.

### Immunofluorescence staining

The binding and uptake of Fc-fused Ecrg4 into HEK293 T cells were determined with fluorescence microscopy. The cells were cultured on poly-l-lysine-coated glass coverslips and transiently transfected with plasmids expressing the indicated membrane proteins. Two days after transfection, the cells were incubated with several Ecrg4–Fc proteins at 37 °C for 2–3 h and then washed three times with PBS. The cells were then fixed with 4% paraformaldehyde in PBS for 10 min and treated with 0.5% Triton X-100 in PBS for 10 min. After blocking with 10% FCS, the coverslips were incubated with an anti-Flag antibody (M2, Sigma) and then with a FITC-conjugated F(ab’)2 donkey anti-human IgG antibody (Jackson ImmunoResearch) and Alexa-594-conjugated goat anti-mouse IgG antibody (Thermo Fisher Scientific). The nuclei were counterstained with Hoechst. Fluorescent images were obtained with an Axio Imager A1 microscope (Carl Zeiss).

### SPR

The binding parameters of 2 × CTLD–Fc to ECRG4 were determined with the ProteOn™ XPR36 Protein Interaction Array System (Bio-Rad). His-tagged ECRG4 peptides were immobilised indirectly with an anti-His-tag antibody. Briefly, an anti-His × 8 antibody (Qiagen) was diluted in 10 mM acetate buffer (pH 4.5) and immobilised on an ethyl(dimethylaminopropyl) carbodiimide/N-hydroxysuccinimide (EDC/NHS)-preactivated ProteOn™ GLM Sensor Chip (Bio-Rad) with amine coupling. The unreacted carboxyl groups on the chip were blocked by the injection of 1 M ethanolamine HCl. The His-tagged Ecrg4 peptides were diluted to 10 μM in HEPES-buffered saline (HBS; 10 mM HEPES (pH 7.4), 150 mM NaCl, 3 mM EDTA, 0.05% surfactant P20; GE Healthcare) and injected for 300 s onto the anti-His-antibody-coated chip for capture. HBS was used as the running buffer in all SPR experiments. For determination of the kinetic parameters of the interaction, different concentrations (250 nM, 125 nM, 62.5 nM, 31.3 nM, 15.6 nM) of the 2 × CTLD–Fc fusion were injected. The injection time was 60 s, and the dissociation time was 120 s. The data were processed with ProteOn™ Manager software. All kinetic parameters, such as the dissociation rate constant (ka), dissociation rate constant (kd), and equilibrium dissociation constant (Kd), were calculated with the Langmuir model.

### Flow cytometry

Cells were treated with 20 μg/mL Fc fusion proteins. After the cells were washed, fixed, and permeabilised, they were stained with FITC-conjugated F(ab’)2 donkey anti-human IgG antibody (Jackson ImmunoResearch). The stained cells were analysed with a FACSCalibur flow cytometer (BD Biosciences).

### RNA preparation and quantitative real-time PCR

Total RNA was extracted with the RNeasy Mini Kit (Qiagen). The first-strand cDNA was synthesised with Transcriptor Reverse Transcriptase (Roche). Real-time PCR was performed with the StepOnePlus Real-Time PCR System (Thermo Fisher Scientific), according to the manufacturer’s protocol. The sample values were normalised to those of the housekeeping gene β-actin with the ΔCt method. The primers used have been described previously^11^. Statistical analyses were carried out with Microsoft Excel software. Data are presented as the mean ± SD. Significant differences were determined using a two-sided Student’s t test. A value of P < 0.05 was considered significant.

### DNA microarray analysis

Total RNA was prepared as described above, amplified and labelled with cyanine 3 using the one-colour Low Input Quick Amp Labeling Kit (Agilent Technologies) according to the manufacturer’s instructions. The labelled cRNA was fragmented and hybridised to the Agilent Whole Mouse Genome Microarray 4 × 44 K ver. 2.0. After the microarrays were washed, they were scanned with an Agilent DNA Microarray Scanner. The intensity value for each scanned feature was quantified with the Agilent Feature Extraction software, which subtracted the background. Agilent GeneSpring GX version 12.1 was used for normalisation, as follows. First, signal intensities <1.0 were set to 1.0. Each chip was then normalised to the 75^th^ percentile of the measurements taken from that chip. Finally, the normalised intensities were log2-transformed. The microarray data have been submitted to the National Center for Biotechnology Information Gene Expression Omnibus (GEO) and are available under accession number GSE106400S.

## Electronic supplementary material

Supplementary Figure 1-5, Original blotting images

## References

[1] Su T, Liu H, Lu S (1998). [Cloning and identification of cDNA fragments related to human esophageal cancer]. Zhonghua Zhong Liu Za Zhi.

[2] Mirabeau O (2007). Identification of novel peptide hormones in the human proteome by hidden Markov model screening. Genome Res.

[3] Li LW (2009). Expression of esophageal cancer related gene 4 (ECRG4), a novel tumor suppressor gene, in esophageal cancer and its inhibitory effect on the tumor growth *in vitro* and *in vivo*. Int J Cancer.

[4] Li W (2010). Overexpression of candidate tumor suppressor ECRG4 inhibits glioma proliferation and invasion. J Exp Clin Cancer Res.

[5] Kujuro Y, Suzuki N, Kondo T (2010). Esophageal cancer-related gene 4 is a secreted inducer of cell senescence expressed by aged CNS precursor cells. Proc Natl Acad Sci USA.

[6] Woo JM (2010). Characterization of changes in global gene expression in the brain of neuron-specific enolase/human Tau23 transgenic mice in response to overexpression of Tau protein. Int J Mol Med.

[7] Gonzalez AM (2011). Ecrg4 expression and its product augurin in the choroid plexus: impact on fetal brain development, cerebrospinal fluid homeostasis and neuroprogenitor cell response to CNS injury. Fluids Barriers CNS.

[8] Podvin S (2011). Esophageal cancer related gene-4 is a choroid plexus-derived injury response gene: evidence for a biphasic response in early and late brain injury. PLoS One.

[9] Kurabi A (2013). Ecrg4 attenuates the inflammatory proliferative response of mucosal epithelial cells to infection. PLoS One.

[10] Lee J (2015). Thrombin-processed Ecrg4 recruits myeloid cells and induces antitumorigenic inflammation. Neuro Oncol.

[11] Moriguchi T (2016). Ecrg4 contributes to the anti-glioma immunosurveillance through type-I interferon signaling. Oncoimmunology.

[12] Podvin, S. *et al*. The Orphan C2orf40 Gene is a Neuroimmune Factor in Alzheimer’s Disease. *JSM Alzheimers Dis Relat Dement* **3** (2016). PMC5157699 27990492

[13] Ozawa A, Lick AN, Lindberg I (2011). Processing of proaugurin is required to suppress proliferation of tumor cell lines. Mol Endocrinol.

[14] Dang X, Podvin S, Coimbra R, Eliceiri B, Baird A (2012). Cell-specific processing and release of the hormone-like precursor and candidate tumor suppressor gene product, Ecrg4. Cell Tissue Res.

[15] Tadross JA (2010). Augurin stimulates the hypothalamo-pituitary-adrenal axis via the release of corticotrophin-releasing factor in rats. Br J Pharmacol.

[16] Baird A (2012). Cell surface localization and release of the candidate tumor suppressor Ecrg4 from polymorphonuclear cells and monocytes activate macrophages. J Leukoc Biol.

[17] Podvin S (2015). Esophageal cancer-related gene-4 (ECRG4) interactions with the innate immunity receptor complex. Inflamm Res.

[18] Kitamura T (2003). Retrovirus-mediated gene transfer and expression cloning: powerful tools in functional genomics. Exp Hematol.

[19] Mehta JL, Chen J, Hermonat PL, Romeo F, Novelli G (2006). Lectin-like, oxidized low-density lipoprotein receptor-1 (LOX-1): a critical player in the development of atherosclerosis and related disorders. Cardiovasc Res.

[20] Yoshimoto R (2011). The discovery of LOX-1, its ligands and clinical significance. Cardiovasc Drugs Ther.

[21] Chen M (2000). Increased expression of lectin-like oxidized low density lipoprotein receptor-1 in initial atherosclerotic lesions of Watanabe heritable hyperlipidemic rabbits. Arterioscler Thromb Vasc Biol.

[22] Cao W (2009). Oligomerization is required for the activity of recombinant soluble LOX-1. FEBS J.

[23] Plüddemann A, Neyen C, Gordon S (2007). Macrophage scavenger receptors and host-derived ligands. Methods.

[24] Canton J, Neculai D, Grinstein S (2013). Scavenger receptors in homeostasis and immunity. Nat Rev Immunol.

[25] Adachi H, Tsujimoto M, Arai H, Inoue K (1997). Expression cloning of a novel scavenger receptor from human endothelial cells. J Biol Chem.

[26] Moriwaki H (1998). Ligand specificity of LOX-1, a novel endothelial receptor for oxidized low density lipoprotein. Arterioscler Thromb Vasc Biol.

[27] Li D, Mehta JL (2000). Upregulation of endothelial receptor for oxidized LDL (LOX-1) by oxidized LDL and implications in apoptosis of human coronary artery endothelial cells: evidence from use of antisense LOX-1 mRNA and chemical inhibitors. Arterioscler Thromb Vasc Biol.

[28] Shimaoka T (2001). LOX-1 supports adhesion of Gram-positive and Gram-negative bacteria. J Immunol.

[29] Tamura Y (2003). FEEL-1 and FEEL-2 are endocytic receptors for advanced glycation end products. J Biol Chem.

[30] Olson MA (2015). Discovery of small molecule inhibitors of MyD88-dependent signaling pathways using a computational screen. Sci Rep.

[31] Cominacini L (2000). Oxidized low density lipoprotein (ox-LDL) binding to ox-LDL receptor-1 in endothelial cells induces the activation of NF-kappaB through an increased production of intracellular reactive oxygen species. J Biol Chem.

[32] Chen XP (2007). Oxidized low density lipoprotein receptor-1 mediates oxidized low density lipoprotein-induced apoptosis in human umbilical vein endothelial cells: role of reactive oxygen species. Vascul Pharmacol.

[33] Jeannin P (2005). Complexity and complementarity of outer membrane protein A recognition by cellular and humoral innate immunity receptors. Immunity.

[34] Means TK (2009). Evolutionarily conserved recognition and innate immunity to fungal pathogens by the scavenger receptors SCARF1 and CD36. J Exp Med.

[35] Beauvillain C (2010). The scavenger receptors SRA-1 and SREC-I cooperate with TLR2 in the recognition of the hepatitis C virus non-structural protein 3 by dendritic cells. J Hepatol.

[36] Murshid A, Gong J, Ahmad R, Borges TJ, Calderwood SK (2015). Scavenger receptor SREC-I promotes double stranded RNA-mediated TLR3 activation in human monocytes. Immunobiology.

[37] Murshid A, Gong J, Prince T, Borges TJ, Calderwood SK (2015). Scavenger receptor SREC-I mediated entry of TLR4 into lipid microdomains and triggered inflammatory cytokine release in RAW 264.7 cells upon LPS activation. PLoS One.

[38] Mehta JL, Li DY (1998). Identification and autoregulation of receptor for OX-LDL in cultured human coronary artery endothelial cells. Biochem Biophys Res Commun.

[39] Ohki I (2011). Surface plasmon resonance study on functional significance of clustered organization of lectin-like oxidized LDL receptor (LOX-1). Biochim Biophys Acta.

[40] Fujita Y (2009). Oxidized LDL receptor LOX-1 binds to C-reactive protein and mediates its vascular effects. Clin Chem.

[41] Ohki I (2005). Crystal structure of human lectin-like, oxidized low-density lipoprotein receptor 1 ligand binding domain and its ligand recognition mode to OxLDL. Structure.

[42] Sheng W (2011). Pro-inflammatory cytokines and lipopolysaccharide induce changes in cell morphology, and upregulation of ERK1/2, iNOS and sPLA_2_-IIA expression in astrocytes and microglia. J Neuroinflammation.

[43] Shields RL (2001). High resolution mapping of the binding site on human IgG1 for Fc gamma RI, Fc gamma RII, Fc gamma RIII, and FcRn and design of IgG1 variants with improved binding to the Fc gamma R. J Biol Chem.

