# Supplementary material for: Ecrg4 peptide is the ligand of multiple scavenger receptors
Source: Sci Rep. 2018 Mar 6;8:4048. doi: 10.1038/s41598-018-22440-4 (PMC5840397; doi:10.1038/s41598-018-22440-4)

Supplementary Information for

Ecrg4 peptide is the ligand of multiple scavenger receptors

Tetsuo Moriguchi, Shuji Takeda, Shinzo Iwashita, Kei Enomoto, Tatsuya Sawamura, Uichi Koshimizu, and Toru Kondo

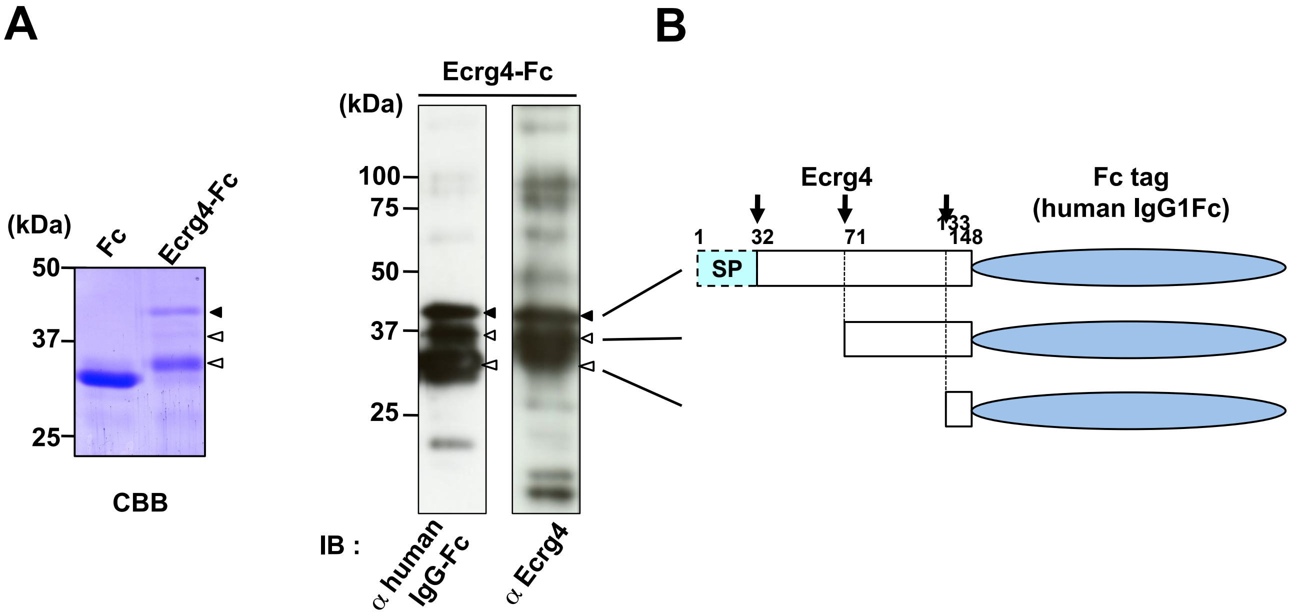

Figure S1. Purified Ecrg4–Fc proteins and their schematic representation. (A) The Fc-fused proteins were purified from the culture supernatants of HEK293T cells transfected with plasmids encoding Ecrg4 fused to human IgG1 Fc (Ecrg4–Fc) or the control IgG1 Fc (Fc). The proteins were detected with Coomassie Brilliant Blue staining (CBB) or immunoblotted (IB) with anti-human IgG Fc antibody (α human IgG-Fc) and anti-ECRG4 antibody (α Ecrg4). (B) Schematic representations of the Ecrg4–Fc proteins. Putative cleavage sites are indicated by arrows. SP: signal peptide. The longest band (black arrowhead) is Fc-fused full-length Ecrg4 after the signal peptide was removed, and the other bands (white arrowheads) are the forms of Ecrg4 processed by furin or thrombin.

A

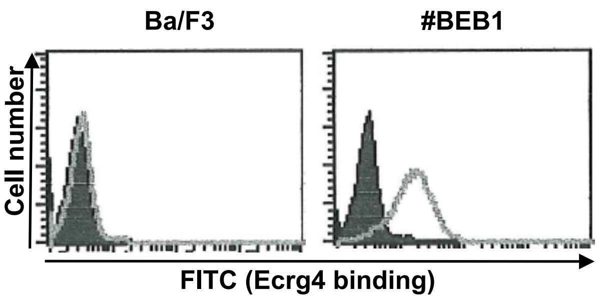

B

ProbeName	GENE_SYMBOL	GENE_NAME	Fold Change
A_52_P150950	Olfm3	olfactomedin 3	489.4609997
A_52_P521507	Trio	triple functional domain (PTPRF interacting)	85.34967769
A_51_P248638	Myoz2	myozenin 2	45.70626103
A_51_P462192	Olr1	oxidized low density lipoprotein (lectin-like) receptor 1	32.64174999
A_55_P1971425	Serpinb9f	serine (or cysteine) peptidase inhibitor, clade B, member 9f	32.53821266
A_55_P2113673	Eml1	echinoderm microtubule associated protein like 1	32.02984919
A_55_P2116714			30.71346123
A_52_P100341	Zfp334	zinc finger protein 334	30.40511203
A_55_P2009077	Sybu	syntabulin (syntaxin-interacting)	30.4047116
A_55_P2174884	Gm2016	predicted gene 2016	29.60166664
A_52_P299771	Bcl2a1c	B cell leukemia/lymphoma 2 related protein A1c	29.45157535
A_55_P2159685	Kcnj3	potassium inwardly-rectifying channel, subfamily J, member 3	29.1293378
A_51_P459320	Gkap1	G kinase anchoring protein 1	28.2026339
A_55_P2042376			28.03625216
A_51_P116064	4921513I03Rik	RIKEN cDNA 4921513I03 gene	27.59967168
A_55_P2033362	Egr2	early growth response 2	27.1892905
A_51_P346132	Rhox9	reproductive homeobox 9	26.94489015
A_55_P1971430	Serpinb9e	serine (or cysteine) peptidase inhibitor, clade B, member 9e	26.70526472
A_55_P1971419	Serpinb9g	serine (or cysteine) peptidase inhibitor, clade B, member 9g	26.25218755
A_51_P322972	Hkdc1	hexokinase domain containing 1	25.74964319

Figure S2. Characterisation of the variant Ba/F3 (#BEB1) cell line, which binds Ecrg4–Fc. (A) Ba/F3 cells and Ba/F3 (#BEB1) cells were incubated with 20 μg/mL Ecrg4–Fc for 30 min on ice. The fluorescence intensities of the bound Ecrg4–Fc are shown. Open curve: staining profile of cells treated with 20 μg/mL Ecrg4–Fc. Closed curve: staining profile of Fc-treated cells. (B) List of the 20 top probe sets upregulated in the Ba/F3 (#BEB1) cells. LOX-1/Olr1 is included in the list.

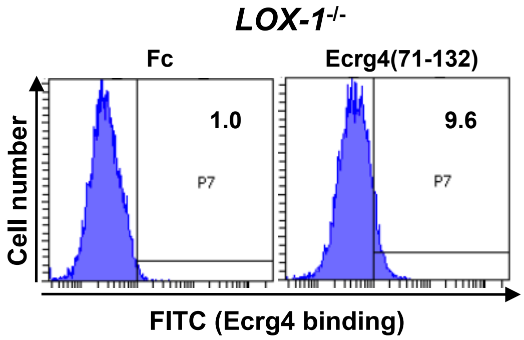

Figure S3. Fluorescence intensities of Ecrg4(71–132) bound to *LOX-1* deficient primary microglia.

Primary microglia from *LOX-1*$^{-/-}$ mice were incubated with Fc(N293A)-fused Ecrg4(71–132) for 1 h at 37°C. After microglia were washed, fixed, and permeabilised, Fc(N293A) proteins were detected with flow cytometry on a FACSCanto™ II cell sorter.

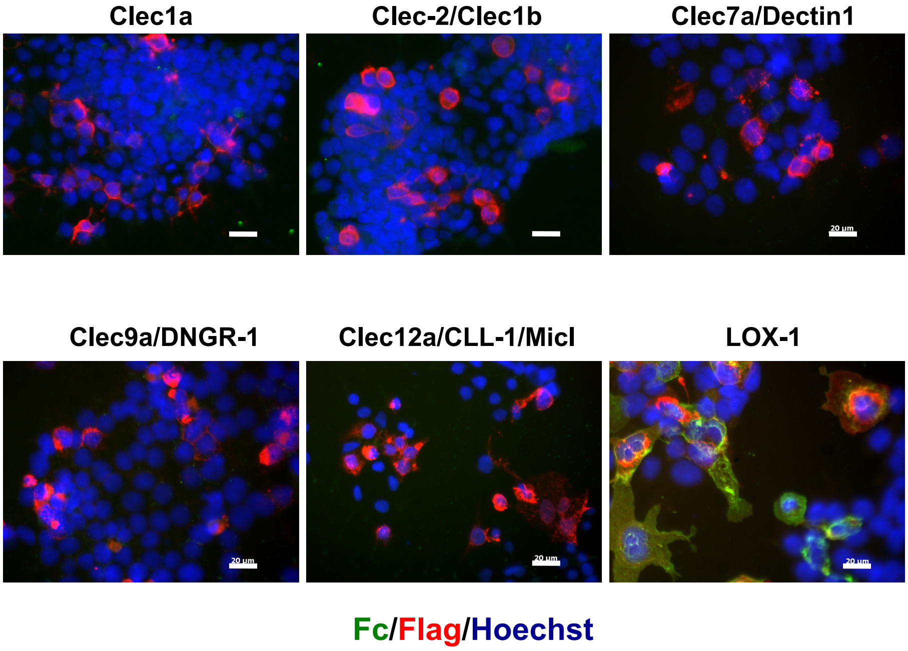

Figure S4. Fluorescence microscopic analysis of HEK293T cells transiently transfected with plasmids encoding several C-type lectin-like receptors. HEK293T cells were transiently transfected with plasmid encoding Flag-tagged mouse Clec1a, Clec2/Clec1b, Clec7a/Dectin1, Clec9a/DNGR-1, Clec12a, and LOX-1. Two days after transfection, the cells were incubated with Ecrg4–Fc protein. The expression of each C-type lectin-like receptor was detected with an anti-Flag antibody (red), and Ecrg4–Fc was measured with a FITC-labelled anti-human IgG Fc antibody (green).

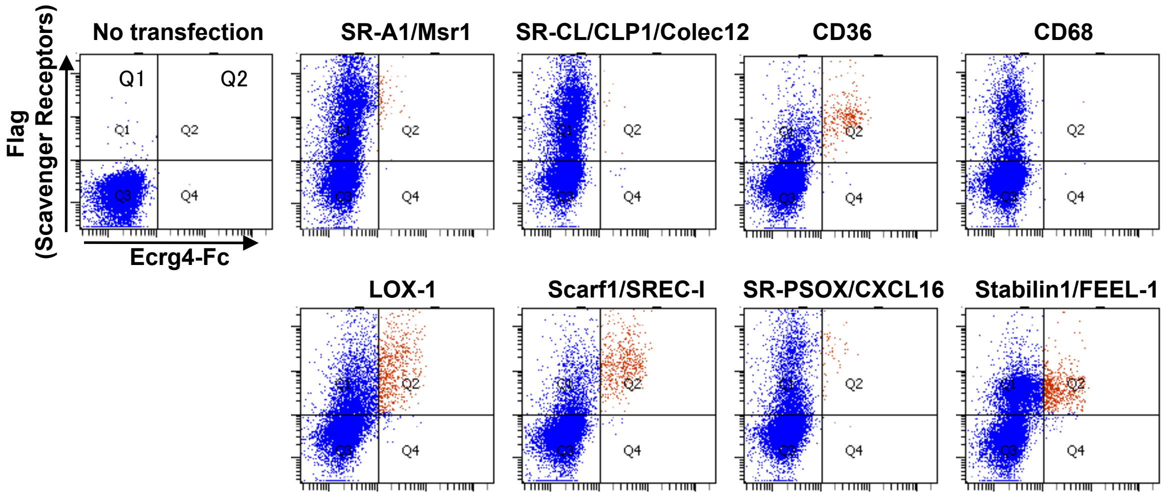

Tested receptors (class X)	Positive ratio
SR-A (class A)	1.7%
SR-CL/CLP1 (class A)	0.3%
CD36 (class B)	20.7%
CD68 (class D)	less than 0.1%
LOX-1 (class E)	22.2%
Scarf1/SREC-I(class F)	29.5%
SR-PSOX/CXCL16 (class G)	2.6%
Stabilin1 (class H)	16.3%

Positive ratio =
(Ecrg4 positive cells (Q2)/Scavenger receptor positive cells (Q1+Q2))

Figure S5. Internalisation of Ecrg4(71–132) by several scavenger receptors. HEK293T cells were transiently transfected with plasmids encoding the indicated Flag-tagged mouse scavenger receptors. Two days after transfection, the cells were incubated with Ecrg4(71–132)–Fc protein, and the expression of the Flag-tagged receptors and the internalised Fc protein were detected with flow cytometry on a FACSCanto™ II cell sorter. Data are representative of three independent experiments. Lower panel: list of the calculated ratios of Ecrg4(71–132) internalisation.

Methods

Animal and plasmids

C57BL/6-background *LOX-1*$^{-/-}$ mice were described previously [1]. Mouse Clec1a (NM_175526), Clec-2/Clec1b (NM_019985), Clec7a/Dectin1 (NM_001309637), Clec9a/DNGR-1 (NM_001205363), and Clec12a/CLL-1/Micl (NM_177686) cDNAs were isolated from the spleens of C57BL/6 mice.

Reference

1. Mehta, J.L. *et al.* Deletion of LOX-1 reduces atherogenesis in LDLR knockout mice fed high cholesterol diet. *Circ Res* **100**, 1634-1642 (2007).

Original blotting images for Fig. 3C.

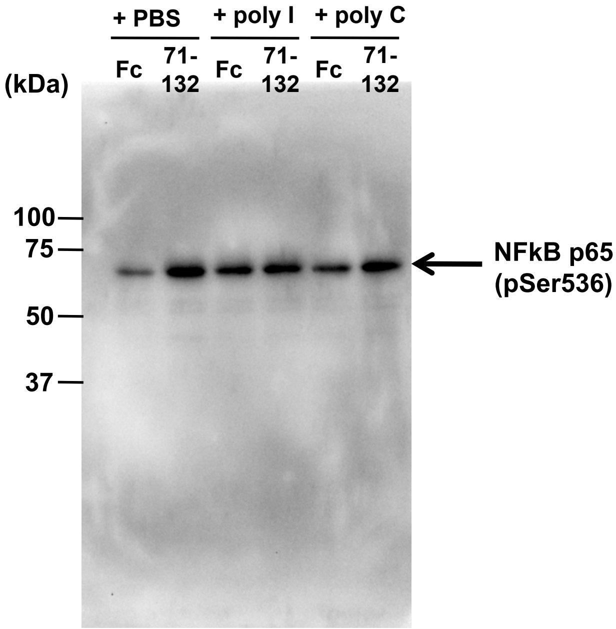

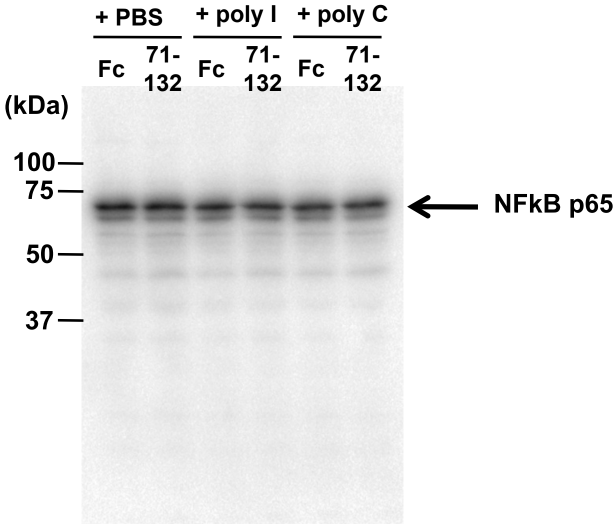

Original blotting images for Fig. 4B.

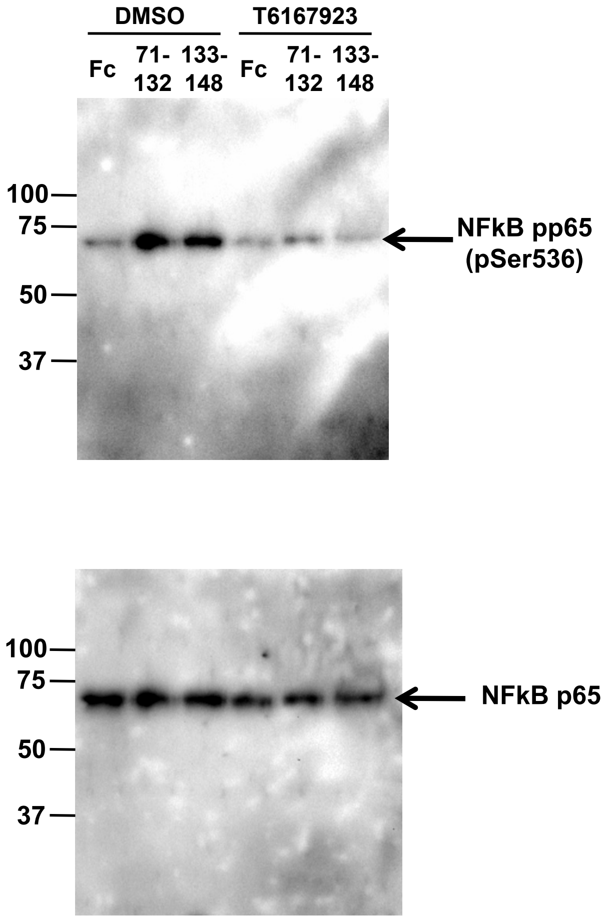

Supplement: Supplementary file 1 — Supplementary Figure 1-5, Original blotting images [file 41598_2018_22440_MOESM1_ESM.pdf]
